# Quantification of the catalytic performance of C1-cellulose-specific lytic polysaccharide monooxygenases

**DOI:** 10.1007/s00253-017-8541-9

**Published:** 2017-12-02

**Authors:** Matthias Frommhagen, Adrie H. Westphal, Roelant Hilgers, Martijn J. Koetsier, Sandra W. A. Hinz, Jaap Visser, Harry Gruppen, Willem J. H. van Berkel, Mirjam A. Kabel

**Affiliations:** 1 Laboratory of Food Chemistry, Wageningen University & Research, Bornse Weilanden 9, 6708 WG Wageningen, The Netherlands; 2 Laboratory of Biochemistry, Wageningen University & Research, Stippeneng 4, 6708 WE Wageningen, The Netherlands; 3 DuPont Industrial Biosciences, Nieuwe Kanaal 7-S, 6709 PA Wageningen, The Netherlands; 4 Fungal Genetics & Technology Consultancy, P.O. Box 396, 6700 AJ Wageningen, The Netherlands

**Keywords:** β-Glucosidase, Lignocellulose, Lytic polysaccharide monooxygenase, Plant biomass, Reducing agent

## Abstract

Lytic polysaccharide monooxygenases (LPMOs) have recently been shown to significantly enhance the degradation of recalcitrant polysaccharides and are of interest for the production of biochemicals and bioethanol from plant biomass. The copper-containing LPMOs utilize electrons, provided by reducing agents, to oxidatively cleave polysaccharides. Here, we report the development of a β-glucosidase-assisted method to quantify the release of C1-oxidized gluco-oligosaccharides from cellulose by two C1-oxidizing LPMOs from *Myceliophthora thermophila* C1. Based on this quantification method, we demonstrate that the catalytic performance of both *Mt*LPMOs is strongly dependent on pH and temperature. The obtained results indicate that the catalytic performance of LPMOs depends on the interaction of multiple factors, which are affected by both pH and temperature.

**Electronic supplementary material:**

The online version of this article (10.1007/s00253-017-8541-9) contains supplementary material, which is available to authorized users.

## Introduction

The enzymatic degradation of plant biomass is considered to be a green and sustainable approach for the production of biochemicals and biofuels. Plant biomass contains a substantial amount of the plant cell wall material lignocellulose, which is resistant for hydrolytic enzymatic degradation. Recently, studies have confirmed that lytic polysaccharide monooxygenases (LPMOs) improve the degradation of lignocellulose (Merino and Cherry 2007; Harris et al. 2010; Forsberg et al. 2011), whereas they have been initially characterized to enhance the decomposition of chitin (Vaaje-Kolstad et al. 2005a; Vaaje-Kolstad et al. 2005b; Vaaje-Kolstad et al. 2010). Based on their amino acid sequences, LPMOs are classified as auxiliary activity (AA) families AA9, AA10, AA11, and AA13 in the Carbohydrate-Active enZyme database (CAZy, Lombard et al. 2014). LPMOs contain a coordinated copper atom and require external electrons for the molecular oxygen-driven oxidation of polysaccharides (Vaaje-Kolstad et al. 2010). Recently, also hydrogen peroxide has been shown to be a co-substrate of LPMOs (Bissaro et al. 2017).

Lignocellulose is mainly composed of cellulose, hemicellulose, and lignin. In addition, free phenolic compounds and phenolic compounds conjugated to hemicellulose are part of lignocellulose. Here, we focus on the catalytic performance of two *Mt*LPMOs from *Myceliophthora thermophila* C1. Both *Mt*LPMOs are active towards cellulose, a homogenous linear polymer that consist of β-(1→4)-linked glucosyl chains. These glucosyl chains interact with each other and form crystalline cellulose fibrils via hydrogen bonds and van der Waals forces (Jarvis 2003; Parthasarathi et al. 2011). In general, cellulose-active LPMOs are able to degrade crystalline cellulose regions by oxidizing the β-(1→4)-linkages at either the C1 or C4 position or at both of these positions (Forsberg et al. 2011; Phillips et al. 2011; Quinlan et al. 2011).

Until now, LPMOs have been characterized for their catalytic mechanism, C1-/C4-regioselectivity, substrate specificity, protein structure, and external electron donation systems (Vaaje-Kolstad et al. 2005b, 2010; Phillips et al. 2011; Westereng et al. 2011; Quinlan et al. 2011). However, the impact of pH and temperature on the catalytic performance of LPMOs has received little attention. One reason is the difficulty of reliable quantification of released C1- and C4-oxidized gluco-oligosaccharides, which differ in their degree of polymerization (DP) (Westereng et al. 2016; Frommhagen et al. 2017b). In addition, commercial standards for oxidized gluco-oligosaccharides are not available. Another reason is that pH and temperature also influence the redox and stability properties of reducing agents (Kracher et al. 2016).

In general, electrons for LPMOs can be provided by multiple sources, such as phenolic compounds (ascorbic acid, gallic acid, lignin) (Vaaje-Kolstad et al. 2010; Westereng et al. 2015; Frommhagen et al. 2016), cellobiose dehydrogenase (CDH) (Langston et al. 2011), photosynthetic pigments, and light-driven water oxidation (Bissaro et al. 2016; Cannella et al. 2016). Also beneficial for the oxidative activity of LPMOs is the co-operation with other enzymes such as GMC-oxidoreductases (glucose-methanol-choline-oxidase/dehydrogenase) or polyphenol oxidases (Kracher et al. 2016; Frommhagen et al. 2017a). Obviously, the synergy with other enzymes increases the complexity of analyzing the pH- and temperature-dependent catalytic properties of LPMOs.

In this research, we describe a procedure for quantifying the catalytic performance of *Mt*LPMO9B and *Mt*LPMO9D that oxidize cellulose at the C1 position in which the *Mt*LPMO9B domain is appended to a CBMI (carbohydrate binding module I), whereas *Mt*LPMO9C is composed of the LPMO domain only. Incubation of the released gluco-oligosaccharide products with β-glucosidase from almonds results in a mixture of gluconic and cellobionic acid, which can be quantified by high-performance anion exchange chromatography. The newly developed method was further applied to study the catalytic performance of *Mt*LPMO9B and *Mt*LPMO9D as a function of pH and temperature. Using either ascorbic acid or 3-methylcatechol as electron donor, it is demonstrated that the oxidative cleavage capacity of both *Mt*LPMOs mainly depends on their operational stability, the pH of the reaction, and the pH-dependent characteristics of the reducing agents. We also describe the use of circular dichroism to investigate the conformational stability of both *Mt*LPMOs as a function of temperature and changes in the secondary structure are discussed in more detail.

## Methods

### Enzyme expression, production, and purification

The strain *M. thermophila* C1 was deposited at the Russian Collection of Microorganisms [VKM] of the Russian Academy of Sciences (Accession No.: VKM F-3500-D) (Emalfarb et al. 1998; Visser et al. 2011). *Mt*LPMO9B was produced and purified as previously described (Frommhagen et al. 2016). The homologous expression of *Mt*LPMO9D (UniProt: KY924631) was performed by using a low protease/low (hemi)cellulose-producing *M. thermophila* C1 strain (Punt et al. 2010; Visser et al. 2011). The *Mt*LPMO9D-containing crude enzyme preparation from the fermentation broth was dialyzed against a 10 mM potassium phosphate buffer (pH 7.0). *Mt*LPMO9D was purified from the dialyzed enzyme preparation by using an ÄKTA-Explorer preparative chromatography system (GE Healthcare, Uppsala, Sweden). The absorbance was continuously monitored at 220 and 280 nm. The protein content of *Mt*LPMO9D-containing fractions was determined as previously described using a BCA Protein Assay Kit (Frommhagen et al. 2015). *Mt*LPMO9D was purified in five subsequent chromatographic steps (see the Supplementary Material for details).

### Enzymes, carbohydrates, and reducing agents

Regenerated amorphous cellulose (RAC) was prepared from Avicel PH-101 as previously described (Zhang and Lynd 2004; Frommhagen et al. 2015). d-Glucose, d-gluconic acid, ascorbic acid, and 3-methylcatechol were purchased from Sigma-Aldrich (Steinheim, Germany). d-Cellobionic acid ammonium salt was obtained from Toronto Research Chemicals (Toronto, Ontario, Canada). Almond β-glucosidase was purchased from Sigma-Aldrich and had, according to the supplier’s information, a specific activity of 6 U mg^−1^ lyophilized powder. Commercial cellulase mixtures Celluclast 1.5 L and Novozym 188 were obtained from Novozymes A/S (Bagsværd, Denmark).

### Catalytic performance of *Mt*LPMO9B and *Mt*LPMO9D: β-glucosidase-assisted quantification

Regenerated (RAC) amorphous cellulose was suspended in a 50 mM ammonium acetate buffer (pH 5.0) to a concentration of 2.8 mg mL^−1^ and incubated with *Mt*LPMO9B (3 mg of protein mg^−1^ substrate) in the absence or presence of ascorbic acid (1 mM). The incubation times and temperatures differed between the experiments performed and are indicated in each figure caption. Samples were incubated in a head-over-tail Stuart rotator (Bibby Scientific, Stone, UK) at 20 rpm. After incubation, all samples were heated for 10 min at 100 °C in a water bath (TW20 Water Bath, JULABO GmbH, Seelbach, Germany) and afterwards cooled down to room temperature (20 °C). Subsequently, all samples were centrifuged (15 min, 15,000×*g*, 4 °C) and 250 μL of the supernatant was dissolved in 750 μL of a 50 mM ammonium acetate buffer (pH 5.0). Finally, 1 U of β-glucosidase was added to each sample. All samples were incubated for 20 h at 37 °C under continuous shaking at 750 rpm (ThermoMixer Comfort, Eppendorf, Hamburg, Germany). After incubation, samples were cooled on ice, centrifuged (1 min, 15,000×*g*, 20 °C), and analyzed by using high-performance anion exchange chromatography (HPAEC). The same protocol as described above was used to determine pH and optimal temperature of *Mt*LPMO9B and *Mt*LPMO9D with the following modifications: (i) RAC was incubated with either *Mt*LPMO9B (3.0 μg of protein mg^−1^ substrate) or *Mt*LPMO9D (1.5 μg of protein mg^−1^ substrate) in the absence or presence of ascorbic acid (1 mM) or 3-methylcatechol (1 mM); (ii) all incubations were performed in a McIlvaine buffer at pH 3.0, 4.0, 5.0, 6.0, 7.0, and 8.0, respectively; and (iii) the pH of all supernatants was adjusted to pH 5.0 prior to β-glucosidase addition. Therefore, 250 μL McIlvaine buffer with a corresponding molarity (50 mM) and a pH between 3.0 and 8.0 was added to 250 μL supernatant until the final pH of the mixture reached precisely pH 5.0. Afterwards, 500 μL McIlvaine buffer (50 mM, pH 5.0) containing 1 U β-glucosidase was added to each sample, which yielded a total volume of 1 mL. All incubations of RAC with and without enzyme addition were performed in triplicate. Samples were diluted 10 times prior to HPAEC analysis. All incubations and experiments were performed without the addition of copper.

### Enzymatic hydrolysis of cellulose obtained from the incubation of RAC with *Mt*LPMO9B

The total cellulose (or RAC) hydrolysis was performed by using a previously described method (Cannella et al. 2016) with the following modifications. Samples (triplicates), obtained from the incubation of RAC with *Mt*LPMO9B in the presence and absence of ascorbic acid, were heated for 10 min at 100 °C in a water bath and cooled down to room temperature (20 °C) afterwards. Samples were then centrifuged (15 min, 15,000×*g*, 4 °C) and the complete supernatant was removed. The remaining pellet was suspended in 1000 μL of a 75 mM ammonium acetate buffer (pH 5.0) and stirred vigorously. Subsequently, Celluclast 1.5 L (0.9%, *w*/*v*) and Novozym 188 (0.18%, *w*/*v*) were added to each sample. All samples were incubated at 50 °C for 20 h. After incubation, samples were centrifuged (15 min, 15,000×*g*, 4 °C) and the supernatant was diluted 20 times prior to HPAEC analysis.

### HPAEC analysis of mono- and oligosaccharides

d-Glucose, gluconic acid, cellobionic acid, and (oxidized) oligosaccharides were analyzed by HPAEC with pulsed amperometric detection (PAD) using a Dionex ICS-5000 system (Sunnyvale, CA, USA) as described previously (Frommhagen et al. 2015) with the following modification. The temperature of the auto sampler was set to 6 °C. A gradient elution program of 35 min was used for the quantification of C1-oxidized gluco-oligosaccharides. In brief, 0–21 min, linear gradient 0–0.25 M NaOAc; 21–25 min, linear gradient 0.25–1 M NaOAc; 25–28 min isocratic gradient 1 M NaOAc; followed by equilibration (7 min) of the column with the starting conditions. Gluconic acid and cellobionic acid were used for calibration in a range of 0 to 50 μg mL^−1^.

### RP-UHPLC-UV-ESI-MS analysis of ascorbic acid and 3-methylcatechol

Ascorbic acid (1 mM) and 3-methylcatechol (1 mM) were dissolved in a 50 mM McIlvaine buffer ranging from pH 3.0 to 8.0. These samples were incubated at different temperatures (20, 30, 40, 50, and 60 °C) under continuous shaking at 750 rpm (ThermoMixer Comfort, Eppendorf) for 12 h. Afterwards, all incubates were centrifuged (15 min, 15,000×*g*, 4 °C) and the supernatant was diluted fifty times in the starting eluent A (H_2_O + 1% (*v*/*v*) acetonitrile + 0.1% (*v*/*v*) HOAc) prior to analysis. The samples were analyzed by using an Accela reversed-phase high-performance liquid chromatography (RP-UHPLC) system coupled to electron spray ionization mass spectrometry (Thermo Scientific, San Jose, CA, USA) as described previously (Frommhagen et al. 2017a). The concentration of ascorbic acid and 3-methylcatechol was determined spectrophotometrically, using an UV-VIS-spectrophotometer (CPS-240A, Shimadzu, Kyoto, Japan). Ascorbic acid was quantified by measuring the absorption at 265 nm (Hernanz 1988). The maximum absorbance of 3-methylcatechol was determined at 262 nm at pH 5.0. The use of different pH values during the incubations led to different absorption maxima of measured samples. Therefore, standard calibration curves were created for all six different pH values for both ascorbic acid and 3-methylcatechol in a range of 0 to 1 mM.

### Reduction potential of ascorbic acid and 3-methylcatechol

Voltammetry experiments were performed by using an Autolab PGSTAT100 Potentiostat (Metronohm, Utrecht, The Netherlands). Both ascorbic acid and 3-methylcatechol (1 mM) were dissolved in a 50 mM McIlvaine buffer ranging from pH 3.0 to 7.0. Temperature-dependent measurements (20–50 °C) were conducted by pre-heating samples in a water bath prior to analysis. Samples were analyzed in duplicate. A three-electrode configuration was used consisting of an Ag/AgCl reference electrode, a glassy carbon working electrode, and a platinum plate counter electrode. Scans were made from − 300 to 800 mV with a scan rate of 50 mV s^−1^.

### Structure-based sequence alignment

The available amino acid sequence and 3D structure of *Mt*PMO3* (PDB entry 5UFV) from *M. thermophila* (ATCC 42464) was used to present the structural features of *Mt*LPMO9D which shares a 100% amino acid sequence identity with *Mt*PMO3* (Span et al. 2017). Hence, only *Mt*LPMO9B was aligned with the available amino acid sequence and 3D-structure of *Mt*PMO3*. The final alignment was obtained by using ESPript (Robert and Gouet 2014).

### Secondary structure analysis

Changes in secondary structure as a function of temperature of *Mt*LPMO9B (0.20 mg mL^−1^) and *Mt*LPMO9D (0.20 mg mL^−1^) were measured by using far-UV circular dichroism. Measurements were performed using a J-715 spectropolarimeter (Jasco Corp., Tokyo, Japan) with a sensitivity of 100 mdeg and a bandwidth of 2 nm. CD spectra of *Mt*LPMO9B and *Mt*LPMO9D were obtained at 20 and 95 °C, respectively. The conformational stability of *Mt*LPMO9B and *Mt*LPMO9D was determined from 20 to 90 °C. The temperature was increased at a rate of 1 °C min^−1^. Protein unfolding was monitored at 202 nm by far-UV CD. Both CD spectra and conformational stability of the *Mt*LPMOs were determined by using a 10 mM potassium phosphate buffer at pH 7.0. All obtained CD spectra of the *Mt*LPMOs have been corrected by subtracting the CD spectra of the buffer. Samples were measured by using quartz cuvettes with an optical path length of 0.1 cm. Secondary structure compositions (%) were calculated by using the online software BeStSel (Kardos and Micsonai 2017). The calculation of the secondary structure composition (%) of *Mt*PMO3* was based on a method described previously (Kabsch and Sander 1983; Touw et al. 2015). In brief, the secondary structure composition was calculated by using the number of amino acids involved in the formation of secondary structures (based on the DSSP file of the PDB entry 5UFV) and the total number of amino acid residues based on the *Mt*PMO3* sequence.

## Results

### Degradation of C1-oxidized gluco-oligosaccharides into gluconic and cellobionic acid

**Fig. 1:**
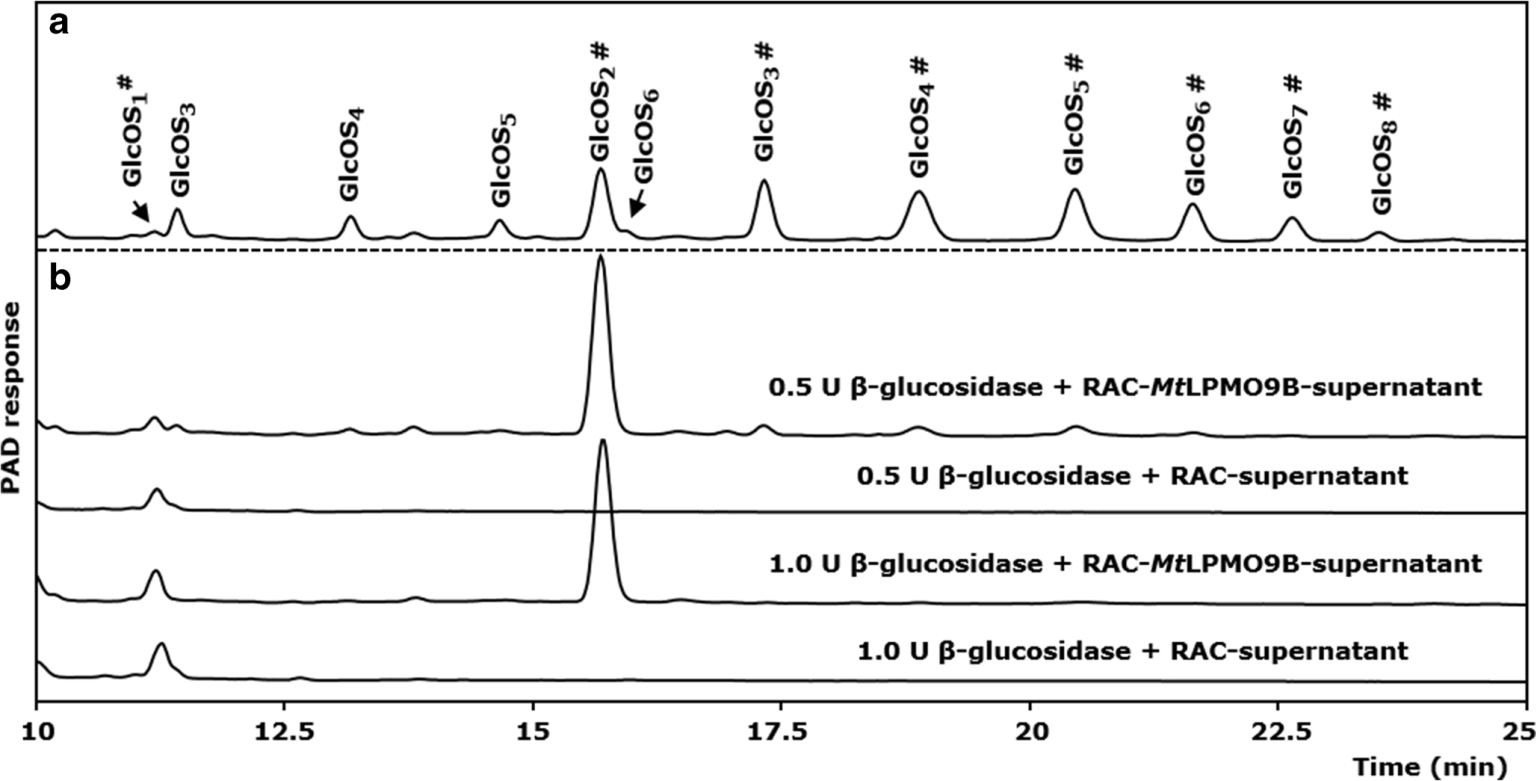
Hydrolysis of released (oxidized) gluco-oligosaccharides with β-glucosidase. **a** HPAEC elution pattern of released non-oxidized (GlcOS_n_) and C1-oxidized (GlcOS_n_^#^) gluco-oligosaccharides after incubation of RAC (2.8 mg mL^−1^) with *Mt*LPMO9B (3 mg g^−1^ substrate) in the presence of 1 mM ascorbic acid. Samples were incubated in a 50 mM ammonium acetate buffer (pH 5.0) at 50 °C for 20 h. **b** Elution profile of the soluble supernatant (250 μL) described under **a**, which was incubated with different amounts of β-glucosidase (0.5 and 1.0 U per sample). Upon addition of 1 U β-glucosidase, all C1-oxidized gluco-oligosaccharides were degraded into cellobionic acid (GlcOS_2_^#^) and glucose (not shown, elution time at 5 min). Only small amounts of gluconic acid (GlcOS_1_^#^) were formed after the addition of β-glucosidase to the supernatant

As previously described (Frommhagen et al. 2016), *Mt*LPMO9B shows activity towards regenerated amorphous cellulose (RAC), which results in the formation of non-oxidized and C1-oxidized gluco-oligosaccharides (Fig. 1a). Hence, *Mt*LPMO9B is characterized as a C1-oxidizing LPMO. *Mt*LPMO9D is characterized as a C1-oxidizing LPMO, based on the product pattern determined by HPAEC (Supplemental Fig. S1) and previous work (Vu et al. 2014; Span et al. 2017).

The ability of β-glucosidase to further degrade the C1-oxidized gluco-oligosaccharides was tested by adding β-glucosidase to the soluble part of the *Mt*LPMO9B-incubated RAC mixture. Under the applied conditions (50 mM ammonium acetate buffer, pH 5.0, 37 °C, 20 h), a complete degradation of the non-oxidized and C1-oxidized gluco-oligosaccharides into glucose (not further analyzed), minor amounts of gluconic acid and cellobionic acid was observed at a dose of 1 U of β-glucosidase (Fig. 1b). In the absence of electron donors, no gluconic or cellobionic acid was formed (Supplemental Fig. S2). The elution profile of β-glucosidase-buffer mixture showed the presence of an unknown compound which elutes at a similar time as gluconic acid (Fig. 1b, Supplemental Fig. S2). Therefore, blanks (β-glucosidase-buffer mixture and RAC incubated with β-glucosidase only) were included for each incubation condition. The area under the curve (AUC) of this blank peak was subtracted from the AUC of the determined peak deriving from the *Mt*LPMO incubation to assure the correct quantification of released gluconic acid (see Supplemental Fig. S2 for details).

### Linearity verification of the β-glucosidase-assisted method

In order to investigate whether the β-glucosidase-assisted method can be used for the quantification of C1-oxidized gluco-oligosaccharides, a dilution series of the soluble part of the *Mt*LPMO9B-incubated RAC mixture (30 h incubation) was prepared and subsequently incubated with 1 U of β-glucosidase. All non-oxidized and C1-oxidized gluco-oligosaccharides in the supernatant were completely degraded into glucose, gluconic acid, and cellobionic acid. The concentration of gluconic acid and cellobionic acid was proportional to the dilution series from 0 to 59.7 nmol mL^−1^ (*R* ^2^ = 0.9945, Supplemental Fig. S4). Hence, the β-glucosidase-assisted method was seen as suitable to determine the total amount of C1-oxidized gluco-oligosaccharides in a range of 0 to 60 nmol mL^−1^.

### Quantification of soluble C1-oxidized gluco-oligosaccharides and insoluble C1-oxidized residues formed by *Mt*LPMO9B

**Fig. 2:**
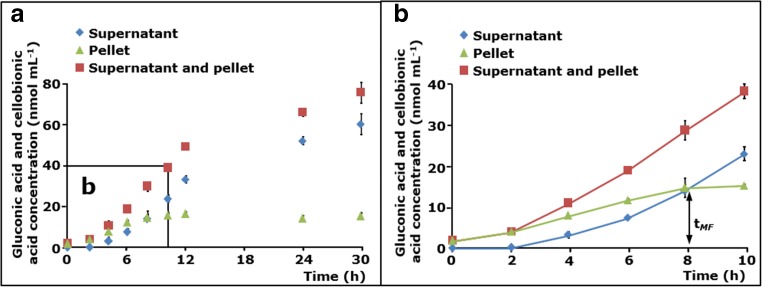
Released gluconic acid and cellobionic acid concentration from RAC incubated with *Mt*LPMO9B measured by using the β-glucosidase-assisted method. The incubation of RAC (2.8 mg mL^−1^) with *Mt*LPMO9B (3 mg g^−1^ substrate) in the presence of ascorbic acid (1 mM) led to the formation of released non-oxidized and C1-oxidzed gluco-oligosaccharides. **a** Soluble fractions obtained from the incubation of RAC with *Mt*LPMO9B were incubated with β-glucosidase (1 U per sample) to yield glucose (not shown), gluconic acid, and cellobionic acid only (diamonds). A modified method based on Cannella and colleagues was used to determine the amount of gluconic acid and cellobionic acid in the insoluble pellet after hydrolysis (triangles) (Cannella et al. 2016). Total amounts of gluconic acid and cellobionic acid obtained from the hydrolysis of soluble and insoluble C1-oxidized gluco-oligosaccharides from RAC incubated with *Mt*LPMO9B in the presence of ascorbic acid (squares). **b** The enlargement highlights the formation of gluconic acid and cellobionic acid from RAC incubated with *Mt*LPMO9B in the first 10 h. The time point (*t*_MF_ *)*, at which the concentration of soluble and insoluble C1-oxidized gluco-oligosaccharides is identical was determined at 8 h

The β-glucosidase-assisted method was applied to determine the release of C1-oxidized gluco-oligosaccharides in the soluble fraction that was obtained after RAC was incubated with *Mt*LPMO9B in the presence of 1 mM ascorbic acid over a time period of 30 h. In the first 2 h, no soluble C1-oxidized gluco-oligosaccharides were formed. From 4 to 30 h, the concentration of C1-oxidized gluco-oligosaccharides steadily increased up to 59.5 nmol mL^−1^ (Fig. 2a, b).

In addition to the soluble fraction, the insoluble fraction of the *Mt*LPMO9B-incubated RAC mixture was degraded using a β-glucosidase-enriched cellulolytic enzyme cocktail based on Cannella and colleagues (Cannella et al. 2016). Several enzyme concentrations were tested in trial experiments in order to ensure that the final enzyme dosage used in this work leads to the complete degradation of the insoluble fraction (Supplemental Fig. S3).

Notably, the *Mt*LPMO9B-mediated oxidation of RAC occurred initially after the start of the incubation, but no soluble C1-oxidized gluco-oligosaccharides were formed within the first 2 h of the incubation. The concentration of C1-oxidized gluco-oligosaccharides increased in the insoluble fraction from 0 to 10 h. Between 10 and 30 h, no further changes in the concentration of C1-oxidized gluco-oligosaccharides were determined in the insoluble fraction (Fig. 2a, b). Interestingly, the concentration of C1-oxidized gluco-oligosaccharides in the insoluble fraction was higher compared to the concentration of released C1-oxidized gluco-oligosaccharides in the soluble fraction until 8 h of the incubation. The incubation time point at which the concentration of C1-oxidized gluco-oligosaccharides in the soluble and insoluble residue is identical was defined by the parameter *t*_MF_ (Fig. 2b).

Notably, the concentration of released C1-oxidized gluco-oligosaccharides from the incubation of RAC with *Mt*LPMO9B did not reach the plateau after 30 h of incubation (Fig. 2). Addition of ascorbic acid after the first 24 h did not increase the product formation (Supplemental Fig. S5). In contrast, an extra load of *Mt*LPMO9B increased the product formation. Hence, it was concluded that *Mt*LPMO9B lost part of its catalytic performance during the incubation (Supplemental Fig. S5).

### Catalytic performance of *Mt*LPMO9B and *Mt*LPMO9D as a function of temperature

**Fig. 3:**
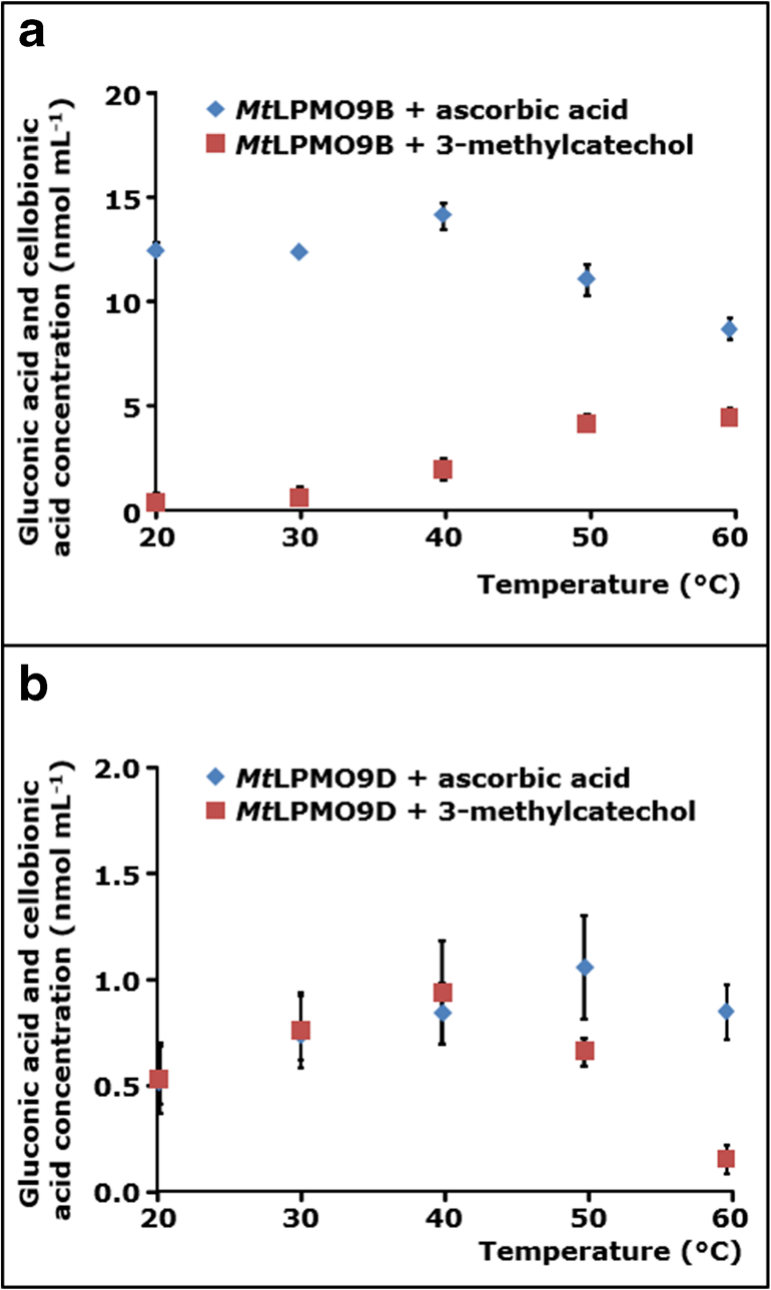
Temperature-dependent activity of *Mt*LPMO9B and *Mt*LPMO9D towards RAC. RAC (1.7 mg mL^−1^) was incubated with **a** *Mt*LPMO9B (3 mg g^−1^ substrate) or **b** *Mt*LPMO9D (1.5 mg g^−1^ substrate) in the presence of ascorbic acid (diamonds) or 3-methylcatechol (squares). Samples were incubated at pH 5.0 for 8 h. Soluble fractions obtained from the incubation of RAC with either *Mt*LPMO9B or *Mt*LPMO9D were further incubated with β-glucosidase (1 U per sample) to yield glucose (not shown), gluconic acid, and cellobionic acid only

As a first step to understand the temperature dependency of *Mt*LPMO9B and *Mt*LPMO9D, the β-glucosidase-assisted method was applied to quantify soluble C1-oxidized oligosaccharides formed at various temperatures between 20 and 60 °C (Fig. 3). The temperature dependency for both *Mt*LPMOs was determined by using one single time point (*t* = 8 h). Therefore, it is possible that this time point lies in the initial rate period or already at the end point of the LPMO reaction, which will be further addressed in the discussion section. *Mt*LPMO9B showed the highest release of C1-oxidized gluco-oligosaccharides at 40 °C in the presence of ascorbic acid, while most C1-oxidized gluco-oligosaccharides were released between 50 and 60 °C in the presence of 3-methylcatechol (Fig. 3a). In comparison, *Mt*LPMO9D showed no significant temperature optimum between 20 and 60 °C and 20 and 50 °C in the presence of ascorbic acid and 3-methylcatechol, respectively (Fig. 3b). In contrast to *Mt*LPMO9D, the catalytic performance of *Mt*LPMO9B between 20 and 40 °C was highly different in the presence of ascorbic acid or 3-methylcatechol.

### Catalytic performance of *Mt*LPMO9B and *Mt*LPMO9D as a function of pH

**Fig. 4:**
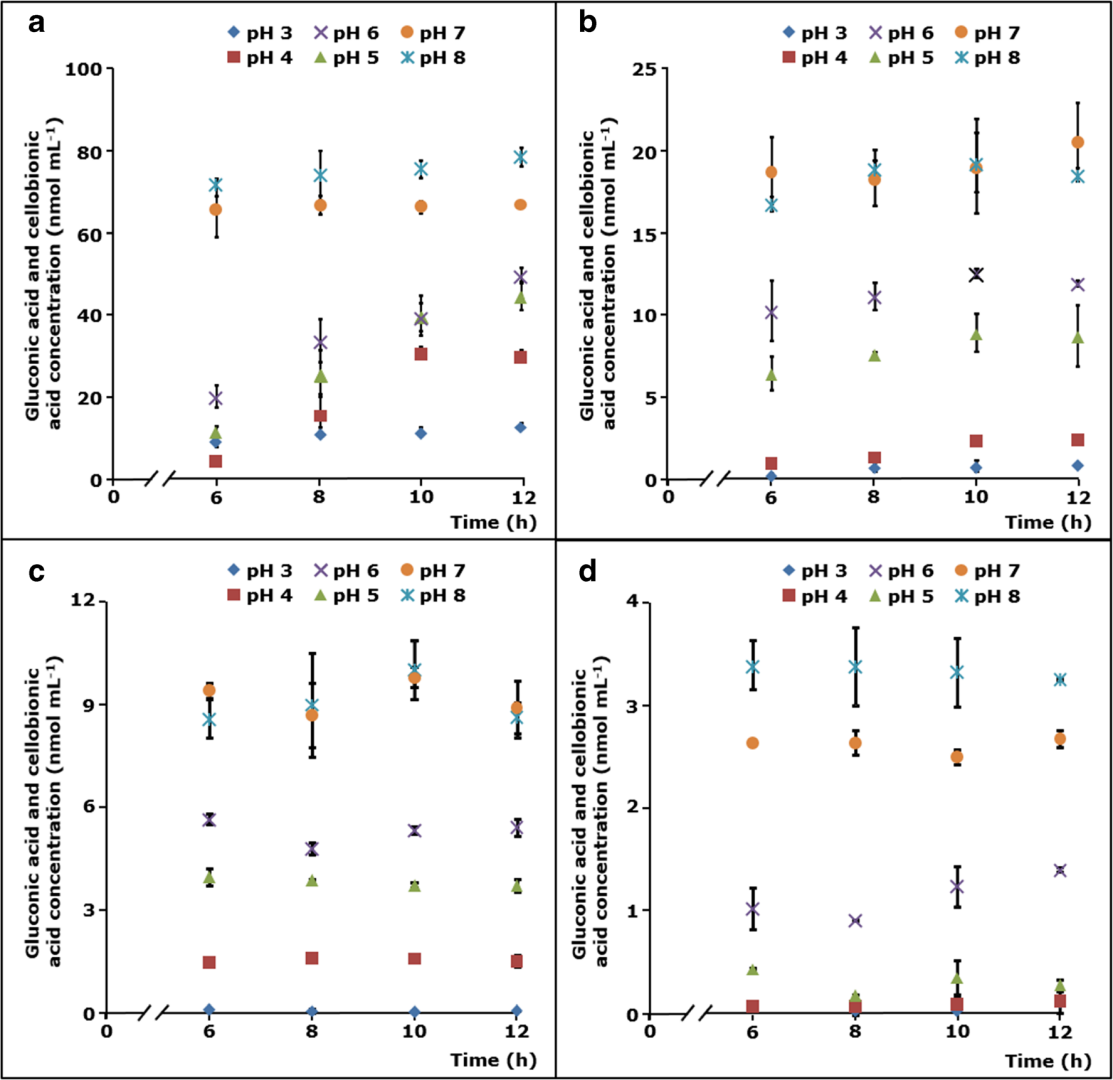
*Mt*LPMO9B and *Mt*LPMO9D activity towards RAC at different pH values. RAC (1.7 mg mL^−1^) was incubated with *Mt*LPMO9B (3 mg g^−1^ substrate) in the presence of **a** ascorbic acid (40 °C) and **b** 3-methylcatechol (60 °C). **c**, **d** Incubation of RAC with *Mt*LPMO9D (1.5 mg g^−1^ substrate) in the presence of **c** ascorbic acid (50 °C) and **d** 3-methylcatechol (40 °C). Samples were incubated at different pH values (see symbols at the top of the graphs) in a 50 mM McIlvaine buffer. Soluble fractions obtained from the incubation of RAC with either *Mt*LPMO9B or *Mt*LPMO9D were incubated with β-glucosidase (1 U per sample) to yield glucose (not shown), gluconic acid, and cellobionic acid only. Optimal temperatures were determined from the incubation of RAC with either *Mt*LPMO9B and *Mt*LPMO9D in the presence of ascorbic acid or 3-methylcatechol at pH 5.0 for 8 h (Fig. 3)

The above-described incubation of RAC with *Mt*LPMO9B in the presence of ascorbic acid showed an almost linear release of C1-oxidized gluco-oligosaccharides between 6 and 12 h (Fig. 2). Therefore, the effect of pH on the catalytic performance of *Mt*LPMO9B and *Mt*LPMO9D towards RAC was studied within this incubation time frame. Due to the different operational stability of both *Mt*LPMOs, this time frame mainly represents the end point of the reaction of RAC incubated with *Mt*LPMO9B and *Mt*LPMO9D in the presence of ascorbic acid or 3-methylcatechol (Fig. 4). The incubations were performed in the presence of ascorbic acid or 3-methylcatechol at the optimal temperatures determined above (Fig. 3). For both *Mt*LPMOs, an increase in pH enhanced the formation of released C1-oxidized gluco-oligosaccharides regardless of the reducing agent used (Fig. 4). Between pH 3.0 and 6.0, the formation of C1-oxidized gluco-oligosaccharides by *Mt*LPMO9B increased between 6 and 12 h (Fig. 4a, b). At a higher pH of 7.0 and 8.0, the catalytic performance of *Mt*LPMO9B already reached the maximum within the first 6 h and hardly any increase of C1-oxidized gluco-oligosaccharides was determined thereafter (Fig. 4a, b). The latter pattern was also observed for *Mt*LPMO9D for all pH values and no increase in the release of C1-oxidized gluco-oligosaccharides was observed between 6 and 12 h of incubation (Fig. 4c, d).

### Ascorbic acid and 3-methycatechol—pH and temperature effects

**Fig. 5:**
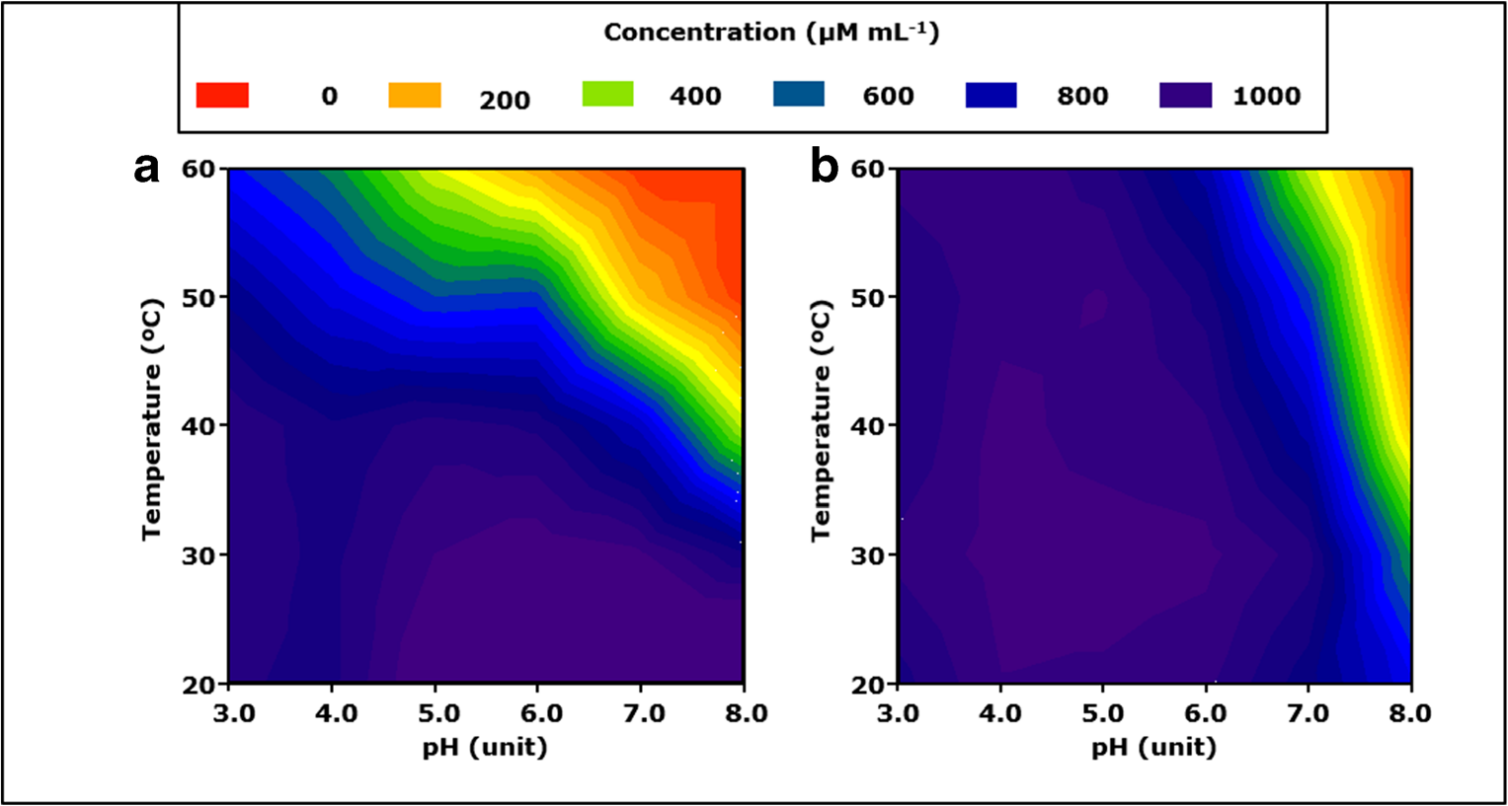
Contour plot of the stability of ascorbic acid and 3-methylcatechol as a function of pH and temperature. Samples containing either **a** ascorbic acid (1 mM) or **b** 3-methylcatechol (1 mM) were incubated at different temperatures (20, 30, 40, 50, and 60 °C) and pH values (3.0, 4.0, 5.0, 6.0, 7.0, and 8.0) for 12 h. The purple and blue regions indicate the highest ascorbic acid and 3-methylcatechol concentrations in the samples after 12 h of incubation. The ascorbic acid and 3-methylcatechol concentrations were analyzed by using UHPLC-MS. The contour plot was obtained by using SigmaPlot 8.0

The observed effects of pH and temperature on the catalytic performance of *Mt*LPMOs raised the question whether and how the reducing agents ascorbic acid and 3-methylcatechol are affected by pH and temperature. Hence, for these two reducing agents, both the stability and their reduction potential were determined at varying pH and temperatures (Figs. 5 and S6). Each reducing agent (1000 μM) was incubated for 12 h and the remaining reducing agent concentration reflected its stability. In addition, standard calibration curves were prepared at each pH that was used to avoid errors by UV quantification, since the pH is known to change the absorption maxima of, e.g., ascorbic acid (Hernanz 1988). Ascorbic acid remained stable (conc. > 800 μM) between pH 3.0 to 8.0 at 20 and 30 °C (Fig. 5a). However, the stability of ascorbic acid decreased at increasing temperature (> 30 °C) and at a higher pH. Above 50 °C, the concentration of ascorbic acid decreased by more than 60% (conc. < 400 μM) at a pH above 5.0 (Fig. 5a).

3-Methylcatechol was stable (conc. > 800 μM) between 20 and 60 °C and at a pH ranging from 3.0 to 6.0 (Fig. 5b). However, 3-methylcatechol became unstable above pH ≥ 7.0, even at low temperatures like 30 °C and above. For instance, the 3-methylcatechol concentration decreased about 50% (conc. 516 μM) when incubated at 30 °C and pH 8.0 for 12 h (Fig. 5b).

The fate of ascorbic acid and 3-methylcatechol during the incubation in the abovementioned conditions was not further investigated. Although not determined in our analysis, ascorbic acid is known to form degradation products such as furfural, 3-hydroxy-2-pyrone, or 2-furoic acid, depending on temperature and pH (Yuan and Chen 1998). In contrast to ascorbic acid, the decline in the 3-methylcatechol concentration was accompanied by the formation of insoluble brown pigments that precipitated after centrifugation (15 min, 15,000×*g*, 4 °C). This pigment formation most likely resulted from the auto-oxidation and polymerization of 3-methylcatechol, which has already been described for other catechol compounds (Yang et al. 2014).

The reduction potentials of ascorbic acid and 3-methylcatechol were measured using cyclic voltammetry. Determined reduction potentials of electron-donating compounds can be used to describe their reducing efficiency on LPMOs. In general, the reducing efficiency of electron-donating compounds increases as the reduction potential decreases. As previously described (Liu et al. 2012), the use of cyclic voltammetry to determine the reduction potential of ascorbic acid is incomplete due to an irreversible reduction of this reducing agent. Therefore, the cathodic peak potential *E*_pc_ was used to illustrate the impact of pH and temperature on the formal reduction potential (*E*°′) of ascorbic acid. A pH increase from 3.0 to 7.0 decreased the cathodic peak potential of ascorbic acid by about 0.25 V at 20 °C, which was similar to the determined difference at 30, 40, and 50 °C (Supplemental Fig. S6a). Thus, ascorbic acid has a stronger reducing efficiency on LPMOs at a higher pH. A temperature increase from 20 to 50 °C decreased the cathodic peak potential of ascorbic acid by about 0.06 V, which was less compared to the pH-dependent decrease (0.25 V) (Supplemental Fig. S6a).

For 3-methylcatechol an increase of the pH from 3.0 to 7.0 led to a decrease of up to 0.22 V of the reduction potential of 3-methylcatechol measured at 20, 30, 40, and 50 °C (Supplemental Fig. S6b). Hence, 3-methylcatechol has a stronger reducing efficiency on LPMOs at a higher pH, just like ascorbic acid. As observed for ascorbic acid, an increase of the temperature from 20 to 50 °C affected the formal reduction potential of 3-methylcatechol only slightly (~ 0.02 V) (Supplemental Fig. S6b).

### Structure-based sequence alignment of *Mt*LPMO9B and *Mt*LPMO9D

*Mt*LPMO9D shares 100% amino acid sequence identity with the recently described *Mt*PMO3*. *Mt*PMO3* is encoded by a gene (gene ID: MYCTH_92668) from *M. thermophila* ATCC 42464, which is a closely related species to *M. thermophila* C1. Furthermore, *Mt*PMO3* was produced via heterologous gene expression in *Neurospora crassa* (FGSC 2489) (Span et al. 2017), whereas *Mt*LPMO9D was obtained from homologous gene expression using the native host *M. thermophila* C1. Recently, Span and colleagues obtained the crystal structure of *Mt*PMO3* (PDB entry 5UFV) (Span et al. 2017), which we used to represent *Mt*LPMO9D in the structure-based sequence alignment (Supplemental Fig. S7). Hence, only *Mt*LPMO9B was aligned with available amino acid sequence and 3D-structure of *Mt*PMO3*. In the alignment, both LPMOs are shown without the signal peptide, while the carbohydrate binding module 1, which is linked to *Mt*LPMO9B, is also not presented. Based on the amino acid sequence, *Mt*LPMO9B has a theoretical molecular weight of 23.8 kDa, whereas the appended CBM-1 has a molecular weight of 6.8 kDa (Frommhagen et al. 2016). The overall amino acid sequence identity of *Mt*LPMO9B (without appended CBM) and *Mt*LPMO9D is 31.8%. Both *Mt*LPMO9B and *Mt*LPMO9D share the β-sheet core that is typical for LPMOs, but differ in their loop regions “loop 2” (L2, Gly16-Val27), “loop short” (LS, Gly108-Trp-126), “loop 8” (L8, Gln162-Pro166), and “long C-terminal loop” (LC, Gly195-Thr228) that are involved in shaping the substrate-binding surface (Li et al. 2012; Wu et al. 2013; Span et al. 2017). The amino acid residues of *Mt*LPMO9B and *Mt*LPMO9D involved in the coordination of the copper are His1-His79-Tyr170 and His1-His75-Tyr169, respectively. Special characteristics of the amino acid sequences of *Mt*LPMO9B and *Mt*LPMO9D are further highlighted in the discussion.

### Conformational stability of *Mt*LPMO9B and *Mt*LPMO9D

**Fig. 6:**
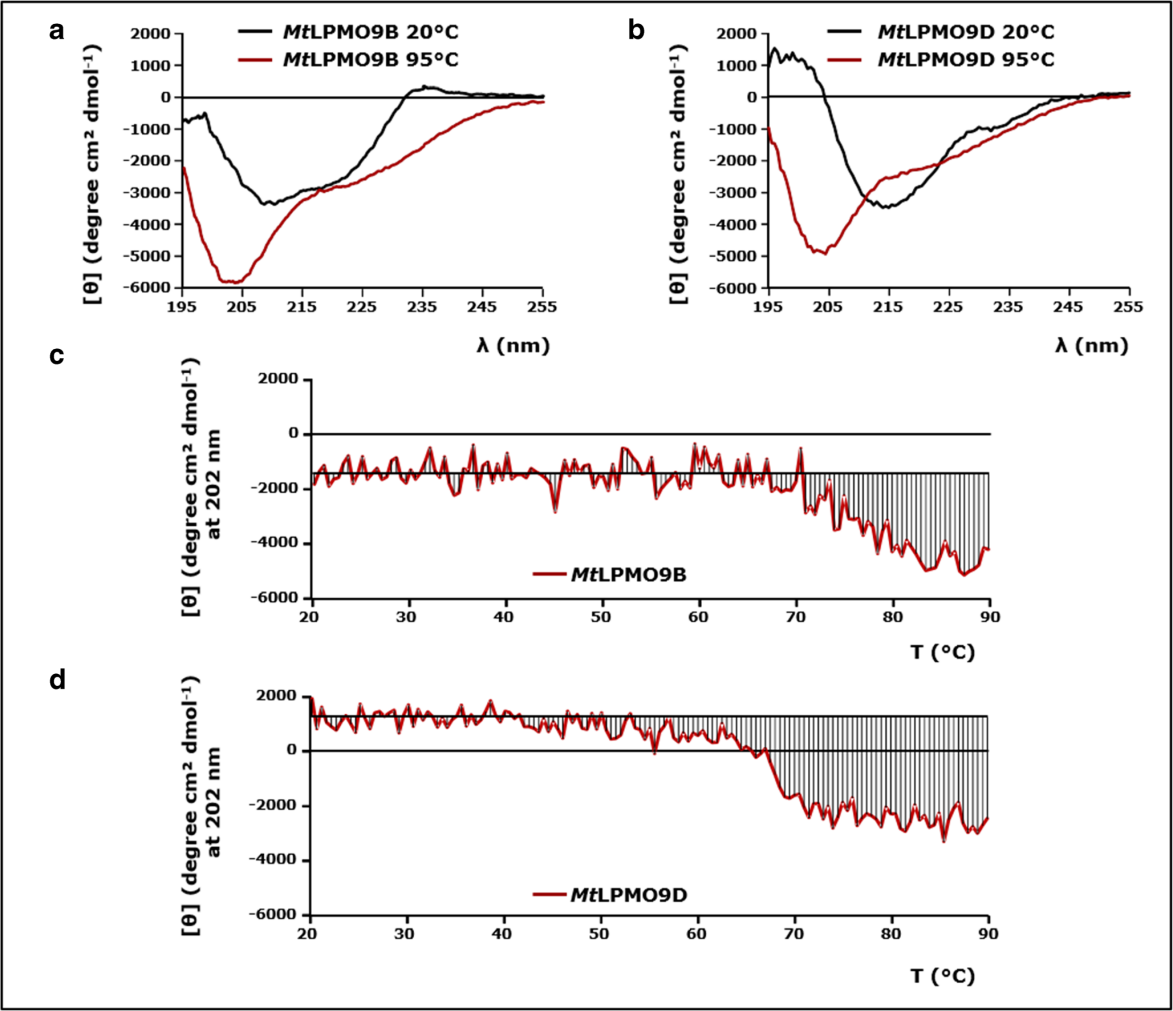
Circular dichroism (CD) spectra and conformational stability as a function of temperature of *Mt*LPMO9B and *Mt*LPMO9D. CD spectra (far UV) of **a** *Mt*LPMO9B (0.20 mg mL^−1^) and **b** *Mt*LPMO9D (0.20 mg mL^−1^) at 20 and 95 °C, respectively. The conformational stability of **c** *Mt*LPMO9B (0.20 mg mL^−1^) and **d** *Mt*LPMO9D (0.20 mg mL^−1^) was determined from 20 to 90 °C. The temperature was increased at a rate of 1 °C min^−1^. Protein unfolding was monitored at 202 nm by far-UV CD. The horizontal line shows the determined mean residue ellipticity [*θ*] (degree cm^2^ dmol^−1^) average between 20 and 40 °C, whereas vertical lines illustrate the deviations from this initial [*θ*] average. The formulas to calculate the mean residue ellipticity [*θ*] were used as described in Greenfield (2007). Both CD spectra and conformational stability of the *Mt*LPMOs were determined by using a 10 mM potassium phosphate buffer at pH 7.0

The conformational stability of *Mt*LPMO9B and *Mt*LPMO9D as a function of temperature was determined by far-UV circular dichroism (CD). The CD spectra recorded at 20 °C (Fig. 6a, b) revealed that both *Mt*LPMOs share a high content of antiparallel β-sheets, whereas the content of α-helices and β-turns is relatively low (Supplemental Fig. S8). The BeStSel method was used because it has been reported to be a reliable tool for the estimation of the secondary structure composition (%) of proteins which contain high amounts of β-structures (Micsonai et al. 2015). It should be noted that the obtained CD spectrum of *Mt*LPMO9B is likely to be influenced by the secondary structure of the appended CBM domain. This CBM1 of *Mt*LPMO9B shares an amino acid sequence identity of 59% with the C-terminal cellulose binding domain (PDB entry 1CBH) of a cellobiohydrolase I (CT-CBH I). It is noteworthy that this CT-CBH I binding domain comprises three antiparallel β-sheets, which is expected to be similar for the CBM I appended to *Mt*LPMO9B (Kraulis et al. 1989).

From monitoring the change in CD signal at 202 nm upon heating, it could be deduced that the *Mt*LPMO9B protein structure was stable in a temperature range between 20 and 70 °C and unfolded gradually at higher temperatures (Fig. 6c). Until now, it is not clear if the gradual change in CD signal above 70 °C is related to the separate unfolding of the *Mt*LPMO9B or appended CBM domain. Figure 6d shows that the CD spectrum of *Mt*LPMO9D already started to change above 40 °C. This gradual change was followed by a sharper transition around 68 °C (Fig. 6d). The impact of temperature on the protein structures was further illustrated by the far-UV CD spectra of *Mt*LPMO9B and *Mt*LPMO9D recorded at 95 °C (Supplemental Fig. S8). Both *Mt*LPMOs comprised less β-sheets and α-helices at 95 °C, whereas the content of β-turns and undefined secondary structures increased. Notably, both proteins still contained a significant amount of secondary structures, such as β-sheets, at this temperature (Supplemental Fig. S8). A reverse temperature decrease from 95 to 20 °C did not lead to refolding of *Mt*LPMO9B or *Mt*LPMO9D to their native state (data not shown).

## Discussion

### Quantification of soluble and insoluble C1-oxidized gluco-oligosaccharides

In this research, we used a β-glucosidase-assisted method to quantify released C1-oxidized gluco-oligosaccharides in order to evaluate the catalytic properties of two C1-oxidizing *Mt*LPMOs (Fig. 1 and Suppl. Fig. S1) (Frommhagen et al. 2016). A comparable method has been described earlier, but was aimed at the quantification of released C1-oxidized chito-oligosaccharides from chitin by the AA10 CBP21 (Loose et al. 2014). Other studies applied multiple enzymes or enzyme cocktails to enable quantification or to ease the analysis of oxidized gluco-oligosaccharides released by LPMOs (Cannella et al. 2012; Müller et al. 2015; Cannella et al. 2016; Westereng et al. 2016). So far, these methods have not been used to discriminate between soluble and insoluble C1-oxidized gluco-oligosaccharides. Additionally, these methods have not been applied to investigate the pH and temperature dependency of LMPOs, which was the aim of the present study. Hereto, it was verified that our β-glucosidase-assisted method is suitable for the quantification of different C1-oxidized gluco-oligosaccharides in a range of 0 to 60 nmol mL^−1^ (Supplemental Fig. S4). The β-glucosidase from almonds, which was used for this method, did not show a significant activity towards cellobionic acid, since we only determined a minor amount of gluconic acid (Fig. 1). In addition, a considerable dose (1 U) of β-glucosidase was needed to cleave released non-oxidized and C1-oxidized gluco-oligosaccharides into glucose, gluconic acid, and cellobionic acid only. It is likely that known inhibitors of β-glucosidases, such as glucose, 1,5-gluconolactone, and gluconic acid, also suppressed the activity of the β-glucosidase from almonds used for this method (Michlmayr et al. 2010; Kara et al. 2014). The developed β-glucosidase-assisted method enables the quantification of C1-oxidized gluco-oligosaccharides whereas the quantification of C4-oxidized gluco-oligosaccharides has not been possible so far.

### Time-dependent cellulose oxidation by *Mt*LPMO9B

We showed that the initial reaction products of *Mt*LPMO9B-mediated oxidation of cellulose are insoluble and that they precede the generation of soluble C1-oxidized gluco-oligosaccharides (Fig. 2). In the incubations with *Mt*LPMO9B (Fig. 2), it took approximately 8 h until more soluble than insoluble C1-oxidized products were formed. This finding is in agreement with the previously described cleavage pattern of *Nc*LPMO9F, which was imaged by atomic force microscopy (AFM) (Eibinger et al. 2014). Although the activity of *Nc*LPMO9F was tested towards crystalline cellulose, AFM imaging showed a degradation of non-soluble cellulose structures, followed by the formation of smaller fragments, which dissolved during the ongoing degradation. We studied *Mt*LPMOs on RAC only, but it can be expected that the time until soluble products are released differs between LPMOs. This expectation is based on the fact that LPMOs show differences in the preference for crystalline and amorphous regions of cellulose (Eibinger et al. 2014; Bennati-Granier et al. 2015; Villares et al. 2017). Obviously, the substrate morphology and the length of glucosyl chains in particular (Jarvis 2003; Zhang and Lynd 2004), will influence the time-dependent release of C1-oxidized gluco-oligosaccharides.

### Catalytic performance of *Mt*LPMO9B and *Mt*LPMO9B as a function of pH and temperature

Here, we show that the pH and temperature dependency of *Mt*LPMO9B and *Mt*LPMO9D is influenced by multiple factors such as type of reducing agent, reducing agent stability, and operational stability of the LPMO enzyme.

As indicated in the “Results” section, incubations of RAC with *Mt*LPMO9B and *Mt*LPMO9D in the presence of both reducing agents at different temperatures have been performed at one single time point (*t* = 8 h, Fig. 3). In addition, the pH dependency of both *Mt*LPMOs has been determined within a time frame of 6 to 12 h, which represents either the linear phase or the end point of the reaction depending on the incubation conditions (Fig. 4). Obviously, both MtLPMOs differ largely in their operational stability. At lower pH values and in the presence of ascorbic acid, *Mt*LPMO9B is more stable but shows a slower catalytic performance towards RAC compared to the incubations at a higher pH. In contrast to *Mt*LPMO9B, *Mt*LPMO9D appears to be very unstable and all results obtained from the incubation of RAC with *Mt*LPMO9D at different pH values represent the end point of the reaction, which must be considered for further interpretation.

At pH 5.0, *Mt*LPMO9B showed a reducing agent-dependent temperature optimum. The catalytic performance of *Mt*LPMO9B was highest at 40 and 60 °C in the presence of ascorbic acid and 3-methylcatechol, respectively (Fig. 3). The decrease in the catalytic performance of *Mt*LPMO9B above 50 °C in the presence of ascorbic acid may have resulted from the accompanying decline in ascorbic acid stability (Fig. 5a). In contrast, 3-methylcatechol was stable over the whole temperature range (20–60 °C) and, consequently, did not influence the temperature optimum of *Mt*LPMO9B (Fig. 5b). It is noteworthy that the reducing efficiency of both reducing agents was hardly affected by the temperature (Supplemental Figs. S6a and S6b). In addition, no alterations in the secondary structure of *Mt*LPMO9B were determined between 20 and 70 °C based of CD measurements at pH 7.0, which illustrates the conformational stability of *Mt*LPMO9B in this temperature range (Fig. 6c).

Nevertheless, highest amounts of determined C1-oxidized gluco-oligosaccharides released from RAC (approximately 80 nmol mL^−1^, Fig. 4a) are several hundred times smaller compared to the used reducing agent concentration (1000 μM, Fig. 5a). Therefore, it is not expected that a minor decline (marked as blue or green in Fig. 5) of the reducing agent concentration during the incubation will significantly decrease the catalytic performance of both *Mt*LPMOs.

In contrast to *Mt*LPMO9B, the catalytic performance of *Mt*LPMO9D significantly decreased at higher temperatures in the presence of 3-methylcatechol but hardly altered in the presence of ascorbic acid (Fig. 3). The small conformational changes of *Mt*LPMO9D that were observed between 40 and 60 °C (Fig. 6d) have not affected the catalytic performance of *Mt*LPMO9D towards RAC in the presence of ascorbic acid. Therefore, the significant decline in the catalytic performance of *Mt*LPMO9D towards RAC in the presence of 3-methylcatechol is not expected to be caused by the conformational stability of the *Mt*LPMO. Since temperature only hardly affected the efficiency of 3-methylcatechol, it can only be speculated if higher temperatures led to an increased formation of, e.g., reactive oxygen species (ROS) and a thereby accompanied decline in the operational stability of *Mt*LPMO9D, which is further explained below (Bissaro et al. 2017).

Next to the temperature dependency, the pH dependency of the catalytic performance of *Mt*LPMOs was investigated. In general, in the presence of both ascorbic acid and 3-methylcatechol, a higher pH enhanced the catalytic performance of *Mt*LPMO9B and *Mt*LPMO9D (Fig. 4). One explanation for this behavior is the decrease in redox potential and thus a stronger reducing power of both electron donors at higher pH (Supplemental Fig. S6). A positive relation between the reducing power of various reducing agents and an improved catalytic performance of LPMOs, such as LPMO-02916, has already been described before (Kracher et al. 2016). Nevertheless, as we do not know the rate-limiting step of catalysis, it is possible that other steps in the reaction cycle contribute to the pH dependency of the activities of both *Mt*LPMO enzymes, such as the impact of pH on protonation reactions that occur during the LPMO-catalyzed substrate oxidation (Walton and Davies 2016). Moreover, it is not known what effect pH and temperature have on the reaction pathway when hydrogen peroxide (H_2_O_2_) acts as co-substrate instead of molecular oxygen (O_2_) (Bissaro et al. 2017).

Interestingly, the increase in the catalytic performance of *Mt*LPMO9B at higher pH values was already at its maximum after 6 h of incubation (Fig. 4a, b). This indicates that *Mt*LPMO9B was inactivated within the first 6 h at pH values above 6.0. As mentioned above, one possible explanation is that the enhanced tendency of reducing agents to donate electrons led to an increased production of ROS. Previous research already reported that the inactivation of LPMOs by ROS can occur within minutes (Bissaro et al. 2017). It was concluded that the formation of ROS in the catalytic center during LPMO activity led to oxidative modification of the catalytic histidine and neighboring residues, which results in LPMO inactivation. In addition, the limited stability of ascorbic acid and 3-methylcatechol above pH 7 at higher temperatures (40–60 °C) also affects the catalytic performance of *Mt*LPMOs (Figs. 4 and 5).

Alternatively, the decreased catalytic performance of both *Mt*LPMOs at low pH values may have been caused by alterations in the coordination of the copper ion in the active site. By revealing the crystal structure of *Ls*AA9 from *Lentinus similis*, Frandsen and colleagues showed that the coordination of the active site histidine (His78) alters at lower pH (3.5) compared to pH 5.5, which possibly results from a protonation of the imidazole (Frandsen et al. 2017a). Such pH-dependent rearrangements of active site histidines will likely affect copper and substrate binding (Frandsen et al. 2017b).

For *Mt*LPMO9D, the incubation conditions were found to be even more critical for optimal performance. This enzyme was inactivated within the first 6 h of incubation at most pH values tested, or alternatively showed hardly any catalytic performance at a low pH (3.0–5.0; Fig. 4c, d).

### Structural features of *Mt*LPMO9B and *Mt*LPMO9D

*Mt*LPMO9B and *Mt*LPMO9D share a sequence identity of 32%. Intriguingly, *Mt*LPMO9D comprises multiple aromatic amino acid residues (1 × Tyr, 2 × Trp, and 1 × Phe) within the L2 loop, whereas other AA9 type LPMOs have been described to contain one or two aromatic residues (Vaaje-Kolstad et al. 2017). In contrast to *Mt*LPMO9D, *Mt*LPMO9B does not contain any aromatic amino acid in the L2 loop. Aromatic amino acids are known to play an important role in shaping the substrate-binding surface, substrate recognition and specificity of LPMOs (Vaaje-Kolstad et al. 2017). Furthermore, it has been indicated that single domain LPMOs may comprise strong substrate-binding abilities (Zeltins and Schrempp 1997; Suzuki et al. 1998; Vaaje-Kolstad et al. 2005b; Nakagawa et al. 2015), whereas LPMO domains appended to CBMs potentially show a decreased substrate affinity (Forsberg et al. 2016; Forsberg et al. 2014b). But, until now, it can only be speculated if the aromatic amino acid residues present in the L2 loop enhance the substrate-binding ability of *Mt*LPMO9D.

Using the developed β-glucosidase-assisted method, we showed that *Mt*LPMO9B releases an overall higher amount of C1-oxidized gluco-oligosaccharides from RAC compared to *Mt*LPMO9D (Fig. 3). Notably, this difference is not only related to the higher enzyme concentration. Whether this higher activity of *Mt*LPMO9B towards RAC is related to the presence of CBM1 and a possibly enhanced substrate binding, can only be presumed. So far, a diverse effect of CBMs on the catalytic performance of LPMOs has been observed and this effect seems dependent on the type of substrate or LPMO used (Forsberg et al. 2014a; Bennati-Granier et al. 2015; Borisova et al. 2015; Crouch et al. 2016).

The structural features of the *Mt*LPMOs are also illustrated in the far-UV CD spectra. Based on the BeStSel method, the calculated secondary structure composition (%) of both *Mt*LPMOs is typical for LPMOs (Supplemental Fig. S8) (Vaaje-Kolstad et al. 2005b; Karkehabadi et al. 2008; Harris et al. 2010). Both *Mt*LPMOs share a high content of antiparallel β-sheets and minor amounts of α-helices and β-turns, but differ significantly in their CD spectra (Supplemental Fig. S8).

It has been shown that proteins which comprise a high amount of β-sheets and a low amount of α-helices have diverse far-UV CD spectral properties, whereas proteins with a high content of α-helices are more similar in their CD spectra (Micsonai et al. 2015). We used the crystal structure of *Mt*PMO3*, which shares a 100% amino acid sequence identity with *Mt*LPMO9D, to demonstrate that the accuracy of the secondary structure prediction based on the obtained CD spectra by using the BeStSel method is in agreement with the actual secondary structure (Span et al. 2017). More details are presented in the Supplemental discussion and Supplemental Fig. S8 .

Interestingly, the secondary structure of *Mt*LPMO9B hardly altered upon heating until 70 °C (Fig. 6c), which is indicative for the high conformational stability of this LPMO. As mentioned in the “Results” section, we do not know to what degree the appended CBM I contributes to the determined structural changes upon heating of *Mt*LPMO9B (Fig. 6). *Mt*LPMO9D, on the other hand, already showed minor structural changes between 40 and 60 °C and lost most of its native structure at 70 °C (Fig. 6). Still, both *Mt*LPMOs maintain a decent amount of structure at 90 °C and both CD spectra of *Mt*LPMOs show a collapse in the signal around 200 nm (Fig. 6a, b). Intriguingly, similar CD spectra with a strong negative band near 205 nm have been shown for poly(Pro)II helix, which might suggest that the *Mt*LPMOs have turned into a kind of fibrillar state during the heat-induced denaturation process (Jenness et al. 1976; Sreerama and Woody 2004).

In this research, we successfully developed a β-glucosidase-assisted method to quantify the release of C1-oxidized gluco-oligosaccharides from RAC incubated with *Mt*LPMO9B and *Mt*LPMO9C. The method was applied to determine the impact of pH and temperature on the catalytic performance of *Mt*LPMOs in the presence of ascorbic acid or 3-methylcatechol. It is concluded that the catalytic performance of *Mt*LPMO9B and *Mt*LPMO9C depends on pH and temperature with a different optimum for each reducing agent.

## Electronic supplementary material

ESM 1 (PDF 436 kb)
