# Supplementary material for: Quantification of the catalytic performance of C1-cellulose-specific lytic polysaccharide monooxygenases
Source: Appl Microbiol Biotechnol. 2017 Dec 2;102(3):1281–95. doi: 10.1007/s00253-017-8541-9 (PMC5778151; doi:10.1007/s00253-017-8541-9)
Supplement: Supplementary file 1 — (PDF 436 kb) [file 253_2017_8541_MOESM1_ESM.pdf]

Applied Microbiology and Biotechnology

Quantification of the catalytic performance of C1-cellulose specific lytic polysaccharide monooxygenases

Matthias Frommhagen[a], Adrie H Westphal[b], Roelant Hilgers[a], Martijn J Koetsier[c], Sandra W A Hinz[c], Jaap Visser[d], Harry Gruppen[a], Willem J H van Berkel[b], Mirjam A Kabel[a]

[a]Laboratory of Food Chemistry, Wageningen University & Research, Bornse Weilanden 9, 6708 WG Wageningen, The Netherlands

[b]Laboratory of Biochemistry, Wageningen University & Research, Stippeneng 4, 6708 WE Wageningen, The Netherlands

[c]DuPont Industrial Biosciences, Netherlands, Nieuwe Kanaal 7-S, 6709 PA Wageningen, The Netherlands

[d]Fungal Genetics & Technology Consultancy, P.O. Box 396, 6700 AJ Wageningen, The Netherlands

Corresponding author:

Mirjam A Kabel, Laboratory of Food Chemistry, Wageningen University & Research, Bornse Weilanden 9, 6708 WG Wageningen, The Netherlands

E-mail: mirjam.kabel@wur.nl

Supplementary Material

Methods

Enzyme purification of *Mt*LPMO9D

First, hydrophobic interaction chromatography (HIC) was applied by loading the *Mt*LPMO9D-containing enzyme preparation on a self-packed Phenyl Sepharose Fast Flow column (450 mL, GE Healthcare, Uppsala, Sweden). The column was pre-equilibrated with 3 column volumes of a 20 mM potassium phosphate buffer (pH 7.8) containing 0.9 M ammonium sulphate. After sample loading, a linear gradient from 0.9 to 0 M ammonium sulphate in a 20 mM potassium phosphate buffer (pH 7.8) was applied at a flow rate of 5 mL min^{-1} over 4 column volumes. All fractions were collected and immediately stored on ice. Peak fractions were, based on UV (280 nm), pooled and concentrated by ultrafiltration (Amicon Ultra, molecular mass cut-off of 3 kDa, Merck Millipore, Cork, Ireland) at 4°C. The concentrated pools were analyzed by SDS-PAGE to determine the *Mt*LPMO9D-containing pool (expected molecular mass 25.4 kDa). The second purification step was applied by using anion exchange chromatography (AEC). The *Mt*LPMO9D-containing fraction was loaded on a Source 30 Q column (50 mL, GE Healthcare) and the column was equilibrated with a 20 mM potassium phosphate buffer (pH 7.8) at a flow rate of 5 mL min^{-1} for 2 column volumes. The elution was performed by using a linear gradient from 0 to 1 M potassium chloride in 20 mM potassium phosphate buffer (pH 7.8) at a flow rate of 5 mL min^{-1} for 10 column volumes. Fractions obtained (10 mL) were immediately stored on ice. Peak fractions were pooled, concentrated and analyzed by SDS-PAGE as described above. In a third purification (HIC) step, the *Mt*LPMO9D-containing pool was loaded on a Phenyl Sepharose Fast Flow column (50 mL, GE Healthcare). The column was pre-equilibrated with a 1.2 M ammonium sulphate in a 20 mM potassium phosphate buffer (pH 7.8). The elution was performed using a linear gradient elution from 1.2 to 0 mM ammonium sulphate in a 20 mM potassium phosphate buffer (pH 7.8) at a flow rate of 5 mL min^{-1} for 4 column volumes. Again, all fractions were immediately stored on ice. The obtained peak fractions were pooled, concentrated and analyzed by SDS-PAGE to determine the *Mt*LPMO9D-containing pool as described above. As a fourth purification step, size exclusion chromatography (SEC) was applied. The *Mt*LPMO9D-containing pool was subjected to a Superdex 75 (250 mL column, GE Healthcare). The equilibration and isocratic elution (2 and 1.5 column volumes, respectively) was performed using a 20 mM potassium phosphate buffer at a flow rate of 3 mL min^{-1}. Fractions were immediately stored on ice. Peak fractions were pooled, concentrated and analyzed by SDS-PAGE to determine the *Mt*LPMO9D-containing pool as described above. In a fifth purification step, the *Mt*LPMO9D-containing fraction was loaded on a Resource Q column (30 x 16 mm internal diameter, GE Healthcare). A 20 mM potassium phosphate buffer (pH 7.0) was used to pre-equilibrate the column. Elution was performed using a linear gradient from 0 to 1 M NaCl in a 20 mM potassium phosphate buffer (pH 7.0) at a flow rate of 6 mL min^{-1} over 20 column volumes and monitored at 220 and 280 nm. All fractions were collected and immediately stored on ice. The fractions of the most abundant peak contained purified *Mt*LPMO9D. Finally, these fractions were pooled, concentrated and stored at -20°C.

Supplementary Material

Methods

Structural features of *Mt*LPMO9B and *Mt*LPMO9D

Based on the predicted secondary structure, the percentage of antiparallel β-sheets was overestimated by about 20% whereas the amount of β-turns was underestimated (-25%) (Supplemental Fig. S8) compared to the actual secondary structure of *Mt*PMO3*. In contrast, the percentage of predicted α-helices corresponded to the amount of α-helices present in *Mt*PMO3*. The deviation between the predicted and the actual secondary structure is likely to result from the limited CD spectral data obtained below 200 nm, which is of importance to obtain a more accurate secondary structure prediction (Kelly et al. 2005). Especially the CD spectrum of *Mt*LPMO9B showed an atypical break below 200 nm, which was also observed if a lower protein concentration (0.1 mg mL^{-1}) was used (data not shown). Possibly, the conditions used (e.g. type of buffer and or pH) were suboptimal for determining a more accurate CD spectrum of the *Mt*LPMOs in the lower wavelength range (< 200 nm). Still, the accuracy of the predicted compared to the actual secondary structure is in agreement with values reported in the literature and, therefore, was further used to determine the conformational stability of the *Mt*LPMOs upon heating (Micsonai et al. 2015).

Supplementary Figures

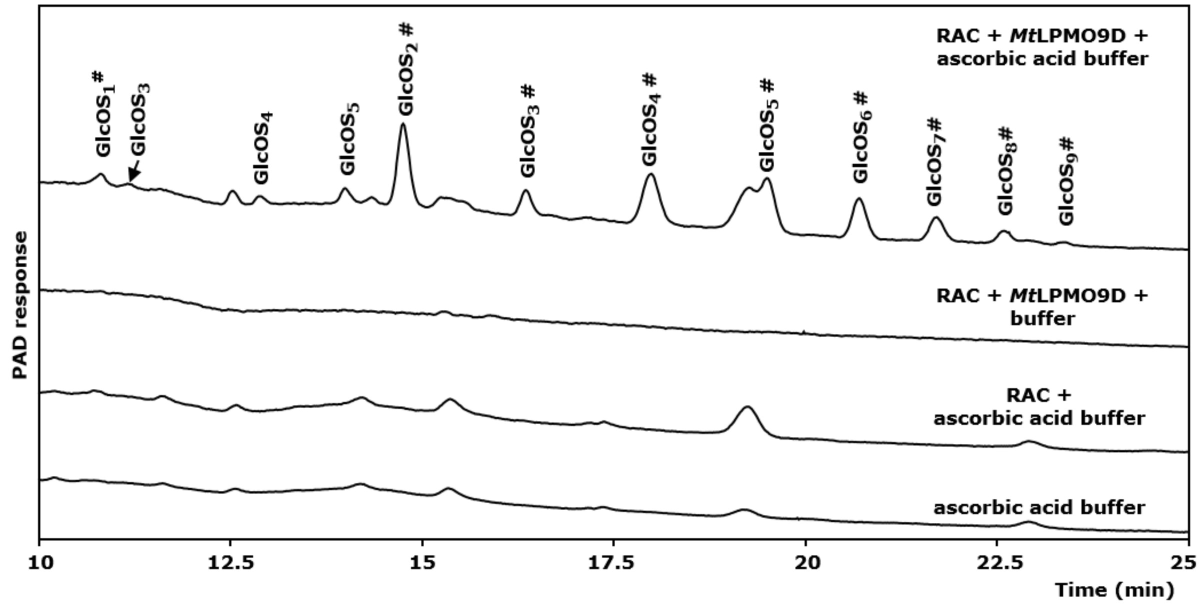

Fig. S1 Activity of *Mt*LPMO9D towards amorphous cellulose. HPAEC elution pattern of regenerated amorphous cellulose (RAC) after incubation with *Mt*LPMO9D (2.5 mg g^{-1} substrate). Nomenclature used: GlcOS$_n$, non-oxidized gluco-oligosaccharides and GlcOS$_n^{\#}$, gluco-oligosaccharides oxidized at the C1 carbon atom. Only in the presence of ascorbic acid, C1-oxidized GlcOS$_n^{\#}$ are formed by *Mt*LPMO9D. Samples were incubated in a 50 mM ammonium acetate buffer (pH 5.0) at 52°C for 24 h in the absence or presence of ascorbic acid (1 mM).

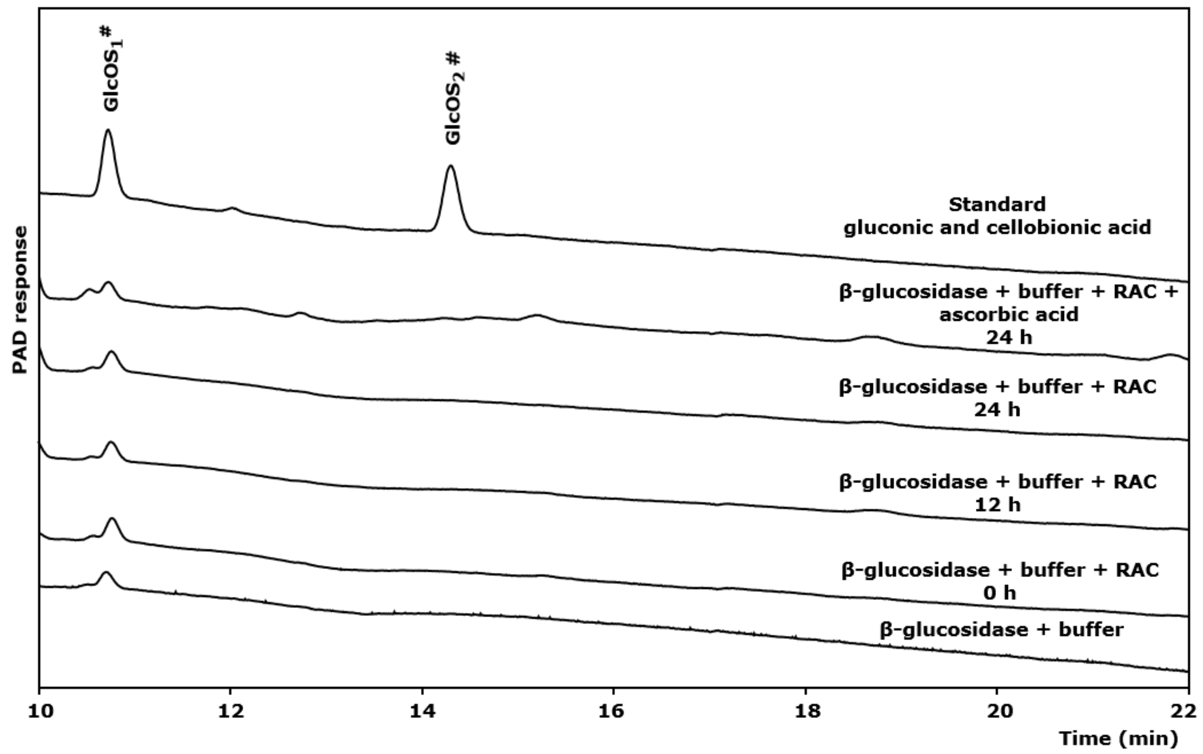

Fig. S2. HPAEC elution pattern of various soluble supernatants incubated with β-glucosidase. First, all samples (buffer only, RAC in buffer (0, 12, and 24 h) and RAC in buffer and in the presence of ascorbic acid (24 h)) were incubated in a 50 mM ammonium acetate buffer (pH 5.0) at 50°C. Subsequently, the obtained soluble supernatants (250 µL) were incubated with β-glucosidase (1.0 U per sample) as described in the Methods section. No oxidized products (gluconic acid and cellobionic acid) were determined if RAC was incubated at the above described conditions. In addition, it is clearly visible that the HPAEC elution pattern of the β-glucosidase-mixture in buffer indicated a peak that elutes at a similar time like gluconic acid.

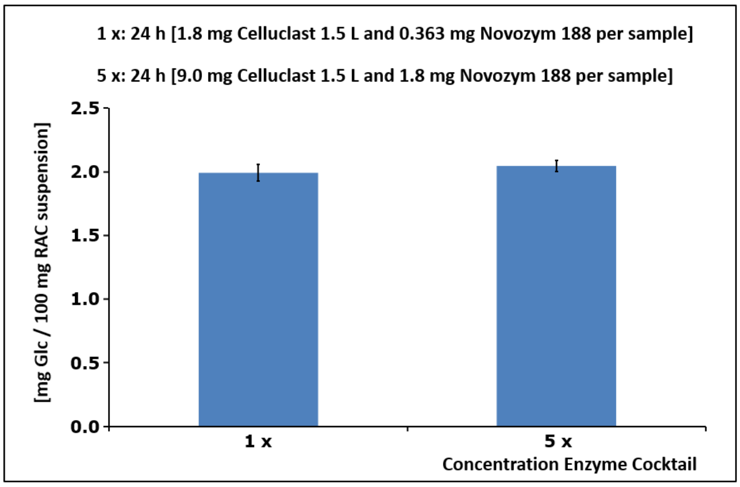

Fig. S3. Total RAC hydrolysis by using Celluclast 1.5l and Novozym 188. The RAC suspension (100 mg) was incubated with two different concentrations of Celluclast 1.5l and Novozym 188 in a 50 mM ammonium acetate buffer (pH 5.0). All samples were incubated at 50°C for 20 h. After incubation, samples were centrifuged (15 min, 15,000 x g, 4°C) and the supernatant was diluted twenty times prior to HPAEC analysis. We did not detect a significant difference between the total amount of glucose released from 100 mg RAC suspension if samples were incubated with an enzyme dosage five times higher than the initial dosage.

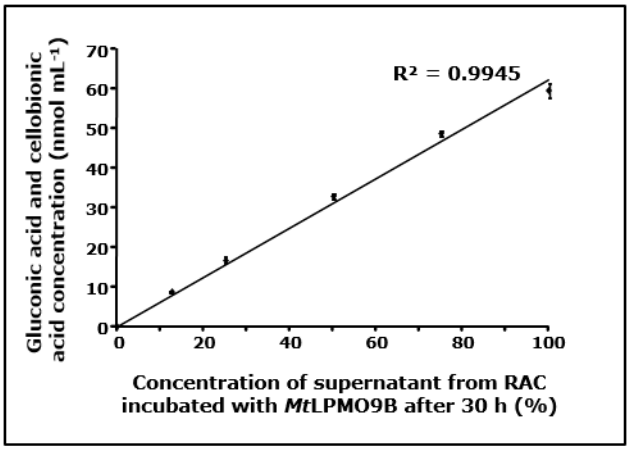

Fig. S4 Verification of the linearity of the β-glucosidase-assisted method. The soluble fraction obtained from the incubation of RAC (2.8 mg mL^{-1}) with *Mt*LPMO9B (3 mg g^{-1} substrate) in the presence of ascorbic acid after 30 h was diluted to obtain samples with varying concentrations of gluconic acid and cellobionic acid. Each diluted sample was incubated with the same amount of β-glucosidase (1 U per sample). The concentration of gluconic acid and cellobionic acid is proportional to the dilutions series (R^2 = 0.9945).

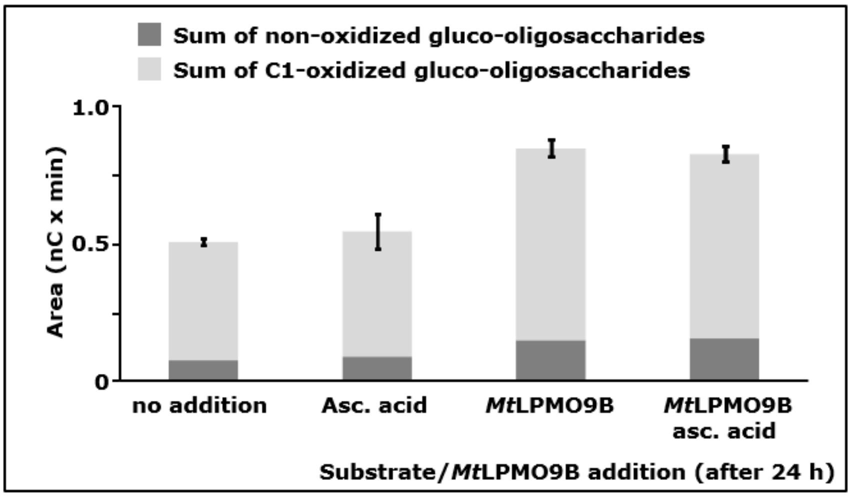

Fig. S5 Activity of *Mt*LPMO9B towards amorphous cellulose. The figure shows the sum of integrated peak areas of released C1-oxidized and non-oxidized gluco-oligosaccharides after incubation of RAC with *Mt*LPMO9B (5 mg g^{-1} substrate) with RAC (1.5 mg mL^{-1}) in the presence of ascorbic acid (1 mM) based on HPAEC. Samples were incubated in 50 mM ammonium acetate buffer (pH 5.0) at 50°C for 48 h. The incubation was interrupted after 24 h and samples were divided into four batches with the following treatments: first batch, no addition of ascorbic acid and no addition of *Mt*LPMO9B; second batch, another addition of 1 mM ascorbic acid but no addition of *Mt*LPMO9B; third batch, no ascorbic acid addition but another addition of *Mt*LPMO9B (5 mg g^{-1} substrate); fourth batch, another addition of 1 mM ascorbic acid and another addition of *Mt*LPMO9B (5 mg g^{-1} substrate). All incubations were performed in duplicate. The standard deviation is represented by error bars, which correspond to one cumulated SD (error bar = ± SDtot; with SDtot = √SD1 2 + SD2 2 +…).

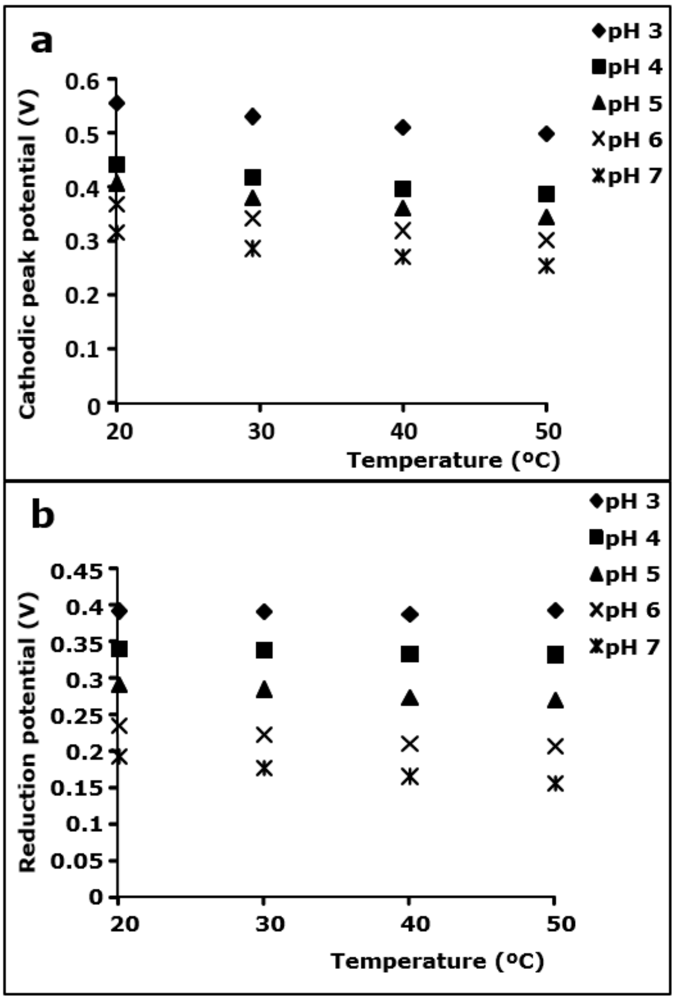

Fig. S6 Redox potentials of ascorbic acid and 3-methylcatechol. The redox potentials of ascorbic acid (1 mM) and 3-methylcatechol (1 mM) were measured at different pH and temperature values by using cyclic voltammetry. **a** – Cathodic peak potential E_{pc} of ascorbic acid at pH 2 to 7 and between 20 and 50°C. The reduction potential (E°') of ascorbic acid was not obtained due to the non-reversible reduction of ascorbic acid during cyclic voltammetry. **b**– Reduction potential (E°') of 3-methylcatechol at **c** pH 2 to 7 and between 20 and 50°C. Standard deviations (not presented) are between 0.001–0.026 (median 0.007) and 0.001–0.004 (median 0.001) for ascorbic acid and 3-methylcatechol, respectively. All samples were measured in duplicate.

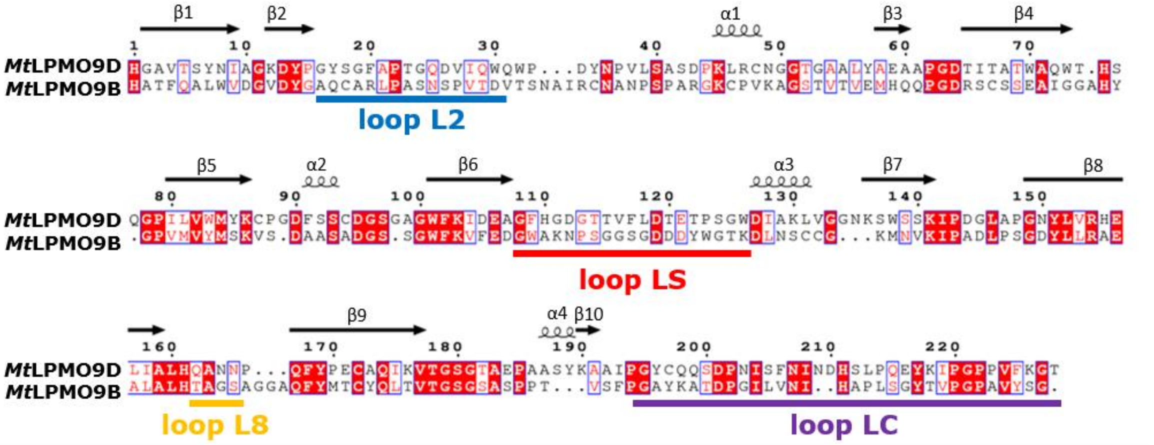

Fig. S7 Structure-based sequence alignment of *Mt*LPMO9B and *Mt*LPMO9D. *Mt*LPMO9D represents the structural features of *Mt*PMO3* (PDB entry 5UFV), because both LPMOs share a 100% amino acid sequence identity (Span et al. 2017). The amino acid sequence of *Mt*LPMO9B was aligned with the amino acid sequence of *Mt*PMO3*. Conserved amino acid residues are presented as white letters on a red background. Amino acid residues that have comparable chemical and physical properties are presented as red letters within blue frames. The secondary structures β-strands (black arrow) and α-helices (black helix) are based on *Mt*PMO3* and shown above the sequences (Span et al. 2017). The amino acid residues of the four loop regions L2 (blue), L8 (yellow), LS (red) and LC (purple) are marked by colored lines below the sequences (Span et al. 2017). Sequences are presented without the signal sequence and start from the N-terminal histidine (His1). The structure-based sequence alignment was obtained by using ESPript (Robert and Gouet 2014).

a

Secondary structure (%)	*Mt*LPMO9B 20°C	*Mt*LPMO9B 95°C	*Mt*LPMO9D 20°C	*Mt*LPMO9D 95°C
**Helix**	8.5	2.8	7.2	4.3
**Antiparallel**	38.3	25.7	32.4	28.5
**Parallel**	0	0	0	0
**Turn**	12.9	16.3	12.7	16.2
**Others**	40.3	55.1	47.7	51

b

Secondary structure (%)	*Mt*PMO3* (5UFV)	*Mt*LPMO9D (BeStSel)
**Helix**	6.3	7.2
**Antiparallel**	26.9	32.4
**Parallel**	0	0
**Turn**	16.8	12.7
**Others**	50	47.7

Fig. S8 Secondary structure composition of *Mt*LPMO9B and *Mt*LPMO9D. **a** The secondary structure composition (%) is based on the obtained CD spectra (far UV) of *Mt*LPMO9B (0.20 mg mL^{-1}) and *Mt*LPMO9D (0.20 mg mL^{-1}) at 20 and 95°C, respectively (see **Figs. 6a** and **6b**). The calculation of the secondary structure composition was based on the BeStSel method (Kardos and Micsonai 2017). **b** Comparison of the predicted secondary structure composition (%) of *Mt*LPMO9D, which was based on the obtained CD spectra and BeStSel method, with the actual secondary structure composition based on *Mt*PMO3* (PDB entry 5UFV) (Span et al. 2017). See Methods for details.
